# Satisfaction with continuity of care from the perspective of older adults who are primary care users and its association with depressive symptoms in Santiago, Chile

**DOI:** 10.3389/fmed.2025.1615842

**Published:** 2025-08-11

**Authors:** Ximena Moreno, Natalia Cornejo

**Affiliations:** ^1^Facultad de Psicología y Humanidades, Universidad San Sebastián, Santiago, Chile; ^2^Independent Researcher, Santiago, Chile

**Keywords:** primary care, older adults, depression, continuity of care, mental health, integrated care

## Abstract

Continuity of care is a fundamental component of integrated care for older adults. Previous research suggests that continuity of care predicts a range of health outcomes in this population and is associated with improved detection and reduction of depressive symptoms. This study aimed to estimate satisfaction with continuity of care among primary care users in Chile and to assess whether satisfaction was associated with a positive screen for depression. Data were collected through face-to-face interviews with 247 community-dwelling older adults enrolled in primary care in Santiago, Chile. Continuity of care and depressive symptoms were measured with the Primary Care Assessment Tool and the 9-item Patient Health Questionnaire, respectively. Linear regression models were used to examine the association between a positive screen for depression and continuity of care, adjusting for sociodemographic characteristics and health status. The mean score for continuity of care was 5.00 (SD = 1.61). A positive screen for depression was independently associated with lower satisfaction with continuity of care (β = −0.94, 95% CI −1.56 to −0.32). Financial hardship (β = −0.50, 95% CI −0.91 to −0.08) and risk of social isolation (β = −0.46, 95% CI −0.88 to −0.05) were also associated with lower satisfaction. The complexity of health needs experienced by older adults, driven by mental health conditions, social and economic vulnerabilities, and multimorbidity, underscores the importance of advancing the implementation of an integrated care model for older adults in primary care in Chile.

## 1 Introduction

Depression is the most prevalent mental health disorder in later life and is the leading mental health-related cause of disability-adjusted life years (1). Despite its importance, a significant proportion of older adults with depression face barriers to accessing mental healthcare (2). From the perspective of older adults, factors that limit access to diagnosis and treatment for depression include the belief that depressive symptoms are a normal part of aging or not severe enough to warrant care, the absence of a regular health provider with whom to talk, and a lack of trust in mental health professionals (3–5). Barriers have also been identified among providers, including insufficient training to address the mental health needs of older adults, the prioritization of physical problems due to time constraints and complexity of needs, and a lower likelihood of referring older adults to psychotherapy (6, 7).

The complexity of health needs in older adults includes a higher likelihood of multimorbidity and disability (8). Moreover, previous evidence suggests that physical health and depression have a mutual influence in later life (9). Pre-existing chronic physical conditions increase the likelihood of developing mental health disorders, and older adults with mental health conditions are more likely to experience a worsening trajectory of physical health (10–12). In older adults, depressive symptoms of any severity are associated with incident disability, and persistent depression increases the risk of mortality (13). To address these complex needs, the World Health Organization (WHO) has proposed integrated care guidelines aimed at preventing, reversing, or delaying adverse physical and mental health outcomes in older adults through community-level interventions led by trained health teams and informed by a comprehensive, context-specific approach (14).

According to the WHO guidelines for integrated care for older people, primary care should be equipped to address the complex health needs of the aging population. This requires the development of a person-centered, integrated approach that can detect and provide effective interventions for both physical and mental health conditions (14). In such an approach, the perspectives and preferences of older adults, along with their personal, family, and community contexts, should be incorporated (15). A recent review of integrated care models for older adults with depression and comorbid conditions identified continuity of care as a core component of these approaches (16). Continuity of care is considered an essential attribute of primary care, defined as the coherence and connectedness of events experienced by users in ways that align with their needs and context (17). It is reflected in the experience of an ongoing relationship with a healthcare team or professional, and of a coordinated, smooth progression of care (18). This definition includes three interrelated dimensions: informational continuity, which refers to knowledge not only of health conditions, but also of preferences, values, and the context of users; management continuity, which refers to the complementary and timely delivery of services by multiple providers in response to changing needs; and relational continuity, which refers to an ongoing relationship with care providers (17, 19).

Previous studies support the positive impact of continuity of care in primary care on multiple health outcomes in older adults. These include greater user satisfaction, adherence to treatment or recommendations, reduced risk of complications among people with chronic conditions, fewer hospitalizations, less frequent use of emergency services, decreased mortality, and lower health care costs (19–23). Research conducted in Canada found that common mental disorders and unmet needs of mental health care were associated with lower satisfaction with primary care and continuity of care among older adults (24, 25). A review of randomized controlled trials concluded that improved continuity of care reduces depressive symptoms and enhances quality of life in this population (12). However, most studies examining the relationship between satisfaction with continuity of care and depression in older adults have been conducted in Europe, North America, or Asia (24–26).

In Latin America, few studies have examined assessments of primary care from the perspective of older adults (27). Most of the available literature has been developed in Brazil, and the findings regarding continuity of care are mixed. In some cases, results suggest that older adults tend to evaluate continuity of care more positively (28–30), whereas in others, they report lower levels of satisfaction with this dimension (31). In Chile, the prevalence of depressive symptoms among older adults who use primary care has been estimated both before and during the COVID-19 pandemic (32, 33). However, to the best of our knowledge, no published study has examined satisfaction with continuity of care or its association with depressive symptoms in this population in Chile. Analyzing these factors may help identify gaps in the implementation of integrated care within primary care settings. Such research could also help assess whether older adults with mental health needs encounter barriers to building trust relationships with health providers or to raising their mental health concerns.

In the Chilean context, 89.8% of people aged 60 years and older are users of the public health system (34). Multidisciplinary teams, including physicians, nurses, midwives, psychologists, physical therapists, occupational therapists, social workers, odontologists, and health technicians, are responsible for delivering preventive and clinical care and for promoting health within defined geographic territories (35). Primary care in Chile is based on a family and community-oriented approach, with a person-centered model and continuity of care as guiding principles (36). Nevertheless, service delivery at the primary care level tends to be fragmented and primarily focused on disease treatment, which undermines continuity of care (37, 38).

The National Plan for Integral Health Care for Older Adults is based on the Comprehensive Family and Community Health Care Model, in which primary care plays a central role (39). At this level, an annual preventive medical evaluation is offered to older adults to monitor their physical, functional, and mental health status and to develop a personalized care plan (39). However, coverage for this evaluation is low, reaching approximately 40% (40). Depression is included among the Explicit Health Guarantees, which ensure access, timeliness, financial protection, and quality of care for people diagnosed with depression (41). Nonetheless, among older adults who screen positive for depression during the preventive medical evaluation in primary care, fewer than 15% have access to diagnosis confirmation (42).

In terms of mental health programs in Chile, current guidelines for the detection and treatment of depression are designed for people aged 15 years and older and lack specific recommendations for older adults (43). Consistent with international literature (2), previous studies have reported that depression is underdiagnosed in older adults in Chile (44, 45). One study that analyzed data from primary care users in Chile found that only 36% of older adults with a positive screen for depression had received a diagnosis (32).

Given the importance of continuity of care as a core component of integrated care for addressing the complex health needs of older adults, this study aimed to estimate the level of satisfaction with continuity of care among community-dwelling older adults using primary care in Chile. Since previous research suggests that continuity of care has a positive impact on health outcomes of older adults with depression (26), we also sought to determine whether satisfaction with continuity of care was associated with a positive screen for depression in the Chilean context.

## 2 Methods

### 2.1 Study design, setting, and participants

The results reported here correspond to the quantitative phase of an ongoing sequential mixed methods study about barriers and facilitators to access diagnosis and treatment for depression among older adults who are primary care users of centers located in the Metropolitan Region of Santiago, in the central macrozone of Chile (46). This region has an aging index of 76.2, and 10% of households are formed by older adults (47). Depressive symptoms among older adults living in this macrozone have a higher prevalence, compared to those living in other regions (32, 48). The study considers the participation of people aged 65 years and older, enrolled in primary care centers of two municipalities in the northern area of Santiago. According to estimations by the Chilean Ministry of Social Development in 2022, the proportion of people experiencing multidimensional poverty in these two municipalities reached 21.2 and 22.4%, respectively (49). The exclusion criteria were: being unable to leave their home or to participate in an interview due to severe health problems. A convenience sample of 253 people was recruited from groups of active users of primary care. Of the total sample, 247 people had complete data on all relevant variables and were included in the analyses. The protocol of the study was approved by the Ethics Committee of the North Metropolitan Health Service. All participants signed an informed consent form before being recruited for the study.

### 2.2 Data collection

A structured face-to-face interview was carried out in a community space near each participant’s home. The interviewers were social science professionals who had experience working with older adults. The interviews lasted an average of 40 min and included questions about sociodemographic characteristics, general health and functional status, and existing validated scales to assess continuity of care and depressive symptoms, as described below.

### 2.3 Measurements

#### 2.3.1 Outcome

Satisfaction with continuity of care was measured with the subscale about continuity of care from the Primary Care Assessment Tools (PCAT), adapted and validated for the adult population in Chile (50). This subscale consists of 14 items about receiving care from the same professional in medical consultations, feelings of being heard by different health professionals in primary care, how much the members of the health team know the user as a patient and a person, and the willingness to move to another primary care, if possible. The response to each item is a 4-point Likert scale, expressing the level of agreement with each item: “definitely not” (1 point), “probably not” (2 points), “probably yes” (3 points), “definitely yes” (4). The average score is calculated, and higher scores indicate a higher level of satisfaction with continuity of care. It has been suggested that a score of 3, which corresponds to the response category “probably yes,” indicates an acceptable level of satisfaction with the attribute. As suggested by the guidelines developed in the Chilean validation of the PCAT, the scores were transformed into a scale of 0 to 10 to allow comparisons with previously published studies (28–30). This standardization is based on the quotient of the mean score obtained in the subscale (0–4) minus 1, and the highest possible score (4) minus 1, multiplied by 10 (50). In this case, 7 points can be considered as an acceptable level of satisfaction with continuity of care (50).

#### 2.3.2 Predictors

Depressive symptoms were measured with the 9-item Patient Health Questionnaire (PHQ-9), developed by Kroenke et al. (51). This instrument has been validated among older adults who are primary care users in Chile (52). The total score of the scale ranges from 0 to 27 points, and a cutoff of 10 or more is considered as a positive screen for major depressive disorder (53).

Sociodemographic variables included were:

‐ Age, as a continuous variable.
‐ Gender, considering male or female.
‐ Level of education, measured as total years of formal education, and collapsed into: “less than primary” (<8 years), “less than secondary” (8–11 years), and “secondary or more” (12 or more years).
‐ Marital status, considering people who were married or with a partner, single, divorced, and widowed.
‐ Financial hardship, assessed with the question: “Considering the total income of your household, including salaries, income from business or paid activities, pensions, rents, money given by other family members, etc., would you say that during the last month this income: was not enough, you had much difficulties; was not enough, you had some difficulty; was just enough, you did not have much difficulty; was enough, you had no difficulties. The first two categories were combined as “financial hardship,” and the last two categories were combined as “no financial hardship.”
‐ Risk of social isolation was assessed with the 6-item Lubben et al. (54). Considering a total score between 0 and 30 points, a cutoff of 12 or less was considered as at risk of social isolation (54).

With respect to health status, the variables considered were:

‐ Self-rated health, measured with the question “In general, would you say that your health is?” The possible responses were: “very good,” “good,” “fair,” “poor,” “very poor.” These categories were collapsed into t: “good” (very good or good), “fair,” and “poor” (poor or very poor).
‐ Functional limitation, measured with the Global Activity Limitation Indicator (55), which assesses limitation in usual activities during the last 6 months. People who report having difficulties, irrespective of the degree, are categorized as with functional limitation.

### 2.4 Statistical analysis

Mean score and standard deviation of the continuity of care subscale of the PCAT were calculated. Mean scores by sociodemographic characteristics and health status were compared using *t*-test for variables with two categories, and ANOVA for variables with more than two categories.

The association between continuity of care and positive screen for depression was estimated with a linear regression model, considering the assessment of continuity of care as the outcome. Finally, a multivariate regression model with the same outcome was estimated to determine if the association with a positive screen for depression held after adjusting for sociodemographic characteristics and health status.

All the analyses were performed with the software Stata 18.0.

## 3 Results

Almost two thirds of the sample (72.87%) were women (Table 1). The mean age was 73.85 years (SD 6.02 years). The most frequent status was married or with a partner (40.49%), followed by widowed (28.74%). Considering proxies of socioeconomic position, 38.47% of the sample had not completed their school education, and 13.77% had not completed primary education. Financial hardship during the last month was reported by 37.25% of the participants. More than half of participants (52.23%) were at risk of social isolation. With respect to health status, 46.96% of the participants had some level of functional limitation, and 42.92% perceived their health as less than good. The frequency of a positive screen for depression was 13.77%.

**TABLE 1:** Characteristics of the sample (*n* = 247).

Variable	*N*	%
**Gender**		
Male	67	27.13
Female	180	72.87
**Marital status**		
Married	100	40.49
Single	31	12.55
Divorced	45	18.22
Widowed	71	28.74
**Years of education**		
<8	34	13.77
8–11	61	24.70
≥12	152	61.54
**Financial hardship**		
Yes	92	37.25
No	155	62.75
**Risk of isolation**		
Yes	129	52.23
No	118	47.77
**Positive screen for depression**		
Yes	34	13.77
No	213	86.23
**Functional limitation**		
Yes	116	46.96
No	131	53.04
**Self-rated health**		
Good	141	57.09
Fair	94	38.06
Poor	12	4.86

The mean score for satisfaction with continuity of care was 5.00 (SD = 1.61; min 0.95, max 9.29). As observed in Table 2, people without a positive screen for depression were more satisfied with continuity of care, in comparison with those with a higher level of depressive symptoms. Additionally, those who were married, without financial scarcity during the last month, and without risk of social isolation, were more satisfied with continuity of care compared with single, divorced or widowed participants, those who reported financial hardship, and people at risk of social isolation.

**TABLE 2:** Mean score and standard deviation for continuity of care, by categories of sociodemographic and health variables.

Variable	Total score	
Gender	Mean (SD)	*p*-value
Male	5.25 (1.67)	0.1303
Female	4.90 (1.58)	
**Education**		
<8	4.58 (1.51)	0.421
8–11	5.13 (1.47)	
12+	5.04 (1.68)	
**Marital status**		
Married	5.16 (1.80)	0.029
Single	4.69 (1.36)	
Divorced	4.87 (1.74)	
Widowed	4.99 (1.33)	
**Financial hardship**		
Yes	4.59 (1.59)	0.0019
No	5.24 (1.58)	
**Risk of social isolation**		
Yes	4.71 (1.42)	0.0029
No	5.31 (1.75)	
**Self-rated health**		
Good	5.04 (1.72)	0.143
Fair	4.94 (1.43)	
Poor	5.00 (1.74)	
**Activity limitation**		
Yes	4.81 (1.62)	0.0861
No	5.16 (1.59)	
**Positive screen for depression**		
Yes	3.97 (1.64)	<0.001
No	5.16 (1.55)	

A positive screen for depression was associated with the level of satisfaction with continuity of care. According to the unadjusted model, people with a positive screen for depression had a score on average 1.19 (95% CI −1.76 to −0.62) points lower on the subscale of continuity of care, compared to those without a positive screen for depression. As depicted in Table 3, in the fully adjusted model, the association held, with an assessment of continuity of care almost 1 point lower among people with a positive screen for depression (β = −0.94, 95% CI −1.56 to −0.32). People who reported financial hardship during the last month, and those who were at risk of social isolation, also had lower scores on continuity of care.

**TABLE 3:** Results of multivariate linear regression.

Variable (reference)	Coefficient	95% CI upper limit	95% CI lower limit	*p*-value
**Positive screen for depression (no)**				
Yes	−0.94	−1.56	−0.32	<0.01
Age	0.02	−0.02	0.05	0.390
**Gender (male)**				
Female	−0.24	−0.73	0.25	0.327
**Level of education (secondary or more)**				
Less than primary	−0.30	−0.91	0.32	0.340
Less than secondary	0.21	−0.27	0.68	0.390
**Marital status (married)**				
Single	−0.09	−0.73	0.56	0.796
Divorced	−0.22	−0.77	0.34	0.444
Widowed	−0.47	−0.57	0.48	0.861
**Financial hardship (no)**				
Yes	−0.49	−0.91	−0.08	<0.05
**Risk of social isolation (no)**				
Yes	−0.46	−0.88	−0.05	<0.05
**Self-rated health (good)**				
Fair	0.28	−0.16	0.72	0.211
Poor	0.61	−0.34	1.56	0.209
**Functional limitation (no)**				
Yes	−0.11	−0.53	0.31	0.611

## 4 Discussion

According to our results, older adults attending primary care centers in the northern area of Santiago reported lower satisfaction with continuity of care compared to previous studies conducted in Brazil, which reported mean scores ranging from 6.50 to 8.04 in the subscale of continuity of care of the PCAT (28–30). Several contextual factors may contribute to explaining these differences. Our study focused on primary care users from densely populated urban areas of Santiago, with more than 10,000 inhabitants per square kilometer - considerably higher than in other parts of the city (56). A report by the Chilean Ministry of Health identified disparities in the distribution of professionals across primary care teams (57), disproportionately affecting areas with higher population density. Moreover, this study was conducted during a period in which primary care and the broader health system were still recovering from the service disruptions and backlogs that occurred during the COVID-19 pandemic (58).

We observed an association between satisfaction with continuity of care and a positive screen for depression, but not with physical or functional health. The prioritization of physical conditions and limited time to discuss mental health concerns have been described as barriers experienced by health providers to address the mental health needs of older adults (6, 7). In our study, although satisfaction with continuity of care was not associated with physical conditions or disability, lower satisfaction was observed among participants with depressive symptoms. This suggests the need to strengthen continuity of care to support the development of long-term, person-focused relationships with regular health providers (20). This is particularly relevant given the strong link between mental health and physical health in later life (9, 11, 12). Depression shapes physical health trajectories and outcomes through complex pathways, including reduced adherence to treatment and medication (13), which is associated with a loss of intrinsic capacity and adverse health outcomes (59–61).

We also found that people at risk of social isolation were more likely to assess continuity of care less favorably. Systematic reviews have shown that social isolation among older adults is associated with adverse health trajectories and outcomes, including frailty, multimorbidity, and mortality (62, 63). Additionally, participants who reported financial hardship were less likely to be satisfied with continuity of care. Previous research suggests that wealth is associated with the maintenance of intrinsic capacity in later life (64), and that economic hardship increases the likelihood of depression in older adults (65).

Our findings underscore the importance of continuity of care for older adults with complex health needs, defined as a combination of chronic conditions, mental health problems, and varying levels of functional limitation, which may be exacerbated by social or economic vulnerabilities (66). A recent review found that older adults with complex needs reported greater satisfaction with continuity of care when fewer providers were involved in their care, and provider skills were more important than meeting always the same person (18). Another review found that continuity of care had a more frequent effect on depression among older adults, and that the level of integration of team members was a key element in achieving this outcome (67). Most studies included in these reviews were conducted in Europe, the United States, or Australia, but evidence from other regions of the world remains scarce.

Our study is the first to examine satisfaction with continuity of care among older adults enrolled in primary care in Santiago, Chile. We also analyzed the association with a positive screen for depression, adjusting for health status and sociodemographic characteristics. These results provide insights into how continuity of care is assessed in this group of users in the post-COVID-19 context. They may also inform comparisons with studies from other geographic and socioeconomic settings or guide future interventions aimed at implementing an integrated care model for older adults in primary care in Chile. A further strength of our study is the use of validated measurement instruments to assess both satisfaction with continuity of care and depressive symptoms, consistent with prior research.

This study has several limitations. We analyzed data from a convenience sample of older adults living in a specific area in Santiago. A large proportion of participants were members of primary care groups or regular attendees of the health center. As a result, our findings are not generalizable to the broader population of older adults using primary care in Chile, particularly those who use services less frequently, reside in rural areas, or live in municipalities with varying number of primary care facilities and professionals per inhabitant. Future studies should incorporate more diverse samples of older adults and include follow-up measurements to assess the impact of continuity of care on depression and health outcomes.

## 5 Conclusion

Among older adults in Santiago, Chile, satisfaction with continuity of care in primary care was lower than reported in previous studies conducted in South America prior to the COVID-19 pandemic. A positive screen for depression was associated with lower satisfaction with continuity of care, even after adjusting for health status and sociodemographic factors. Continuity of care is an essential dimension in addressing the specific demands and expectations of older adults with complex health needs, including mental health problems, social and economic vulnerabilities, multimorbidity, and functional limitations. These results underscore the importance of advancing the implementation of an integrated care model for older adults in primary care in Chile.

## Data Availability

The raw data supporting the conclusions of this article will be made available by the authors, without undue reservation.
